# Using Constrained Factor Mixture Analysis to Validate Mixed-Worded Psychological Scales: The Case of the Rosenberg Self-Esteem Scale in the Dominican Republic

**DOI:** 10.3389/fpsyg.2021.636693

**Published:** 2021-08-19

**Authors:** Zoilo Emilio García-Batista, Kiero Guerra-Peña, Luis Eduardo Garrido, Luisa Marilia Cantisano-Guzmán, Luciana Moretti, Antonio Cano-Vindel, Víctor B. Arias, Leonardo Adrián Medrano

**Affiliations:** ^1^School of Psychology, Pontificia Universidad Católica Madre y Maestra, Santiago de los Caballeros, Dominican Republic; ^2^Faculty of Psychology, Universidad Siglo 21, Córdoba, Argentina; ^3^Faculty of Psychology, Universidad Complutense de Madrid, Madrid, Spain; ^4^Faculty of Psychology, Universidad de Salamanca, Salamanca, Spain

**Keywords:** wording effects, response bias, self-report, factor mixture analysis, method factor, dimensionality assessment, self-esteem, scale validation

## Abstract

A common method to collect information in the behavioral and health sciences is the self-report. However, the validity of self-reports is frequently threatened by response biases, particularly those associated with inconsistent responses to positively and negatively worded items of the same dimension, known as wording effects. Modeling strategies based on confirmatory factor analysis have traditionally been used to account for this response bias, but they have recently become under scrutiny due to their incorrect assumption of population homogeneity, inability to recover uncontaminated person scores or preserve structural validities, and their inherent ambiguity. Recently, two constrained factor mixture analysis (FMA) models have been proposed by Arias et al. (2020) and Steinmann et al. (2021) that can be used to identify and screen inconsistent response profiles. While these methods have shown promise, tests of their performance have been limited and they have not been directly compared. Thus the objective of the current study was to assess and compare their performance with data from the Dominican Republic of the Rosenberg Self-Esteem Scale (*N* = 632). Additionally, as this scale had not yet been studied for this population, another objective was to show how using constrained FMAs could help in the validation of mixed-worded scales. The results indicated that removing the inconsistent respondents identified by both FMAs (≈8%) reduced the amount of wording effects in the database. However, whereas the Steinmann et al. method only cleaned the data partially, the Arias et al. (2020) method was able to remove the great majority of the wording effects variance. Based on the screened data with the Arias et al. method, we evaluated the psychometric properties of the RSES for the Dominican population, and the results indicated that the scores had good validity and reliability properties. Given these findings, we recommend that researchers incorporate constrained FMAs into their toolbox and consider using them to screen out inconsistent respondents to mixed-worded scales.

## Introduction

A common method to collect information in the behavioral and health sciences is the self-report (Weijters et al., 2010; Demetriou et al., 2015; Fryer and Nakao, 2020). Through the self-report a large amount of quantitative information can be collected at low cost that allows generalizations about the population and to obtain highly useful results for society (Demetriou et al., 2015). Among its many uses, the self-report helps to diagnose psychological disorders, know political attitudes, personality characteristics, physical activity and eating habits, working conditions, and perceived quality of services and products, etc. (e.g., Emold et al., 2011; Griffiths et al., 2014; Van Hiel et al., 2016; Schuch et al., 2017; Soto and John, 2017). However, the validity of the self-report is threatened by response biases, particularly those associated with the semantic polarity of the items (Weijters et al., 2013; Plieninger, 2017; Baumgartner et al., 2018; Chyung et al., 2018; Vigil-Colet et al., 2020). These response biases associated with the semantic polarity of the questions are commonly referred to as *wording effects*.

Wording effects are common in data from mixed-worded psychological scales and they have a deleterious effect on the psychometric properties of the instruments’ scores (Swain et al., 2008; Nieto et al., 2021). Wording effects can create spurious dimensions, alter the factor structure of psychological scales, deteriorate model fit, reduce the reliability of the scale scores, alter structural relationships, among other impacts (DiStefano and Motl, 2006; Woods, 2006; Weijters et al., 2013; Savalei and Falk, 2014; Arias et al., 2020; Nieto et al., 2021). Recently, two constrained factor mixture analysis (FMA; Lubke and Muthén, 2007) models have been proposed that can be used to screen individuals who produce response profiles that exhibit strong wording effects (Arias et al., 2020; Steinmann et al., 2021). These strategies can be very useful to preserve the psychometric properties of mixed-worded scales, and can help understand the characteristics of the individuals that produce these biased response profiles. However, because these proposals are very recent, tests of their performance are limited and do not include direct comparisons. Thus, the objective of this study was to examine the performance of these two constrained FMA methods with scores from the Rosenberg Self-Esteem Scale (RSES; Rosenberg, 1965), the most widely used scale to study wording effects (Steinmann et al., 2021). Additionally, a second objective was to perform the first adaptation and validation study of the RSES for the Dominican Republic population, thus showing the benefits of using the constrained FMAs for the validation of mixed-worded psychological scales.

The rest of the Introduction is organized as follows: *first*, we present a brief overview of wording effects, with emphasis on its definition and causes. *Second*, we discuss the limitations of the traditional factor modeling methods that have been used to handle wording effects. *Third*, we describe the characteristics of the two constrained FMAs that haven been proposed. *Fourth*, we present a brief review of the RSES literature, with a special focus on the studies examining wording effects. Finally, we detail the objectives of the present study and its contributions to the literature.

### Wording Effects: Definition and Causes

Wording effects in mixed-worded psychological scales occur when individuals respond inconsistently to questions of the same underlying trait but formulated in a positive sense (in the direction of the trait or characteristic that is to being measured) and in a negative sense (in the opposite direction of the trait or characteristic that is to being measured; Kam and Meyer, 2015). For example, if we want to evaluate whether a person suffers from depression, a positive item would be “I feel sad most of the time” and a negative item would be “I usually feel happy.” Respondents that agree (or disagree) with both sentences would be providing inconsistent answers on a logical level.

Three fundamental causes have been identified for the wording effects in the responses to mixed-worded scales: inattention, acquiescence, and item verification difficulty (Swain et al., 2008; Baumgartner et al., 2018; Nieto et al., 2021). Inattention constitutes a lack of effort on the part of the respondent when reading and answering the questions (Arias et al., 2020), which can lead to answers that do not reflect the person’s real position. For its part, acquiescence constitutes the tendency to agree or disagree with survey items regardless of their content (Kam, 2016). Item verification difficulty refers to cases where people have difficulties in understanding the questions and in selecting the answer option that best reflects their way of thinking or feeling (Swain et al., 2008). These three causes produce the same observed result, which is the logical incongruity in the responses to positive and negative items of the same latent dimension.

### Limitations of Traditional Factor Modeling of Wording Effects

There are numerous factor modeling strategies that have been employed to account for wording effects in data from self-reported psychological scales. Some of the most frequently used have been: correlated traits-correlated uniqueness (CT-CU), correlated traits-correlated methods (CT-CM), correlated traits-correlated methods minus one (CT-C[M-1]), and random intercept item factor analysis (RIIFA), among others (Tomás and Oliver, 1999; Horan et al., 2003; DiStefano and Motl, 2006; Marsh et al., 2010b; Weijters et al., 2013; Gu et al., 2015; Michaelides et al., 2016; Gnambs et al., 2018). These strategies, in particular the CTC(M-1) and RIIFA, have been shown to perform well in accounting for wording effects variance in both simulation and empirical studies (e.g., Geiser et al., 2008; DiStefano and Motl, 2009; Tomás et al., 2013; Savalei and Falk, 2014; Abad et al., 2018; de la Fuente and Abad, 2020; Schmalbach et al., 2020; Nieto et al., 2021). However, even though these traditional factor modeling strategies have been widely used in the psychological literature and have been shown to perform well in accounting for wording effects variance, they have some serious limitations that hinder their applicability. We discuss some of the most important limitations next.

*First*, these traditional factor modeling strategies wrongly assume population homogeneity. Assuming population homogeneity implies that the wording effect applies to all respondents to a certain degree (Steinmann et al., 2021). However, recent results with mixture modeling have shown that the wording effect is primarily the result of a small proportion of individuals, 4 to 20% according to different estimates (Yang et al., 2018; Arias et al., 2020; Steinmann et al., 2021), that respond inconsistently to the positively and negatively worded items of the same scale. In cases such as these the CT-CM or CT-C(M-1), also considered bifactor models, are implausible models that constitute a spurious finding resulting from unaccounted population heterogeneity (Raykov et al., 2019).

*Second*, these factor modeling strategies are not able to recover the unbiased or uncontaminated trait scores of the inconsistent respondents. That is, while they can account for the wording effects variance in the data well, the factor scores of the inconsistent respondents on the substantive traits will remain biased (Nieto et al., 2021). This has important implications regarding the applicability of these models, because if the trait estimates are biased due to the wording effects it can potentially affect important properties of the data, such as reliability, measurement invariance, and the relationships with other constructs (Tomás et al., 2015; Nieto et al., 2021).

*Third*, as shown through Monte Carlo simulation by Nieto et al. (2021) and remarked by other authors (e.g., Reise et al., 2016; Arias et al., 2020; Ponce et al., 2021; Steinmann et al., 2021), scores on wording method factors are difficult to interpret. One of the reasons is because they can arise from different causes (e.g., careless responding, acquiescence, and item verification difficulty), and may behave differently for each, in some cases even providing scores that reflect the standing of the inconsistent participants on the substantive trait of interest. In addition, the same methods (e.g., CT-CM, CT-C[M-1]) are used to model method variance related to wording effects and specific substantive variance related to hierarchical traits (i.e., bifactor models). However, there is no clear way based on these models’ parameter estimates to determine which of these sources of variance the “method factor” is actually capturing or if it is some combination of both (Reise et al., 2016; Arias et al., 2020). As a result of these issues, relationships (or lack thereof) of the method factor scores with other variables are ambiguous at best and are limited in their capacity to enrich our understanding of wording effects and the respondents that exhibit them.

As a result of the aforementioned limitations of the “method factor approach” to addressing wording effects in mixed-worded scales, recently some authors have proposed constrained versions of FMAs that can be used to identify, screen, and study participants that respond inconsistently to positively and negatively worded items corresponding to the same scale. We outline these proposals next.

### Constrained Factor Mixture Analysis for Wording Effects Data Screening

Mixture models, also known as *hybrid* models, are a group of statistical techniques that are useful to explore unobserved population heterogeneity. Populations are said to be heterogeneous when they are composed of clusters of individuals, also known as subpopulations. If the source of the heterogeneity is observed (e.g., gender, age, etc.), the subpopulations are called *groups*, and group membership is known for each participant. Data of this type can be modeled using multiple-group analyses. If, on the other hand, the source of the heterogeneity is unobserved the subpopulations are called *latent classes* and class membership for participants must be inferred from the data. Traditionally, data from populations where unobserved heterogeneity is suspected have been modeled with latent class analysis (LCA) techniques. More recently, however, hybrid models that relax the within class restrictions of LCA have been developed. One of these hybrid techniques is FMA (Lubke and Muthén, 2007).

Factor mixture analysis combines the common factor model (Thurstone, 1947) and the classic latent class model (Lazarsfeld and Henry, 1968) and includes a single categorical and one or more continuous latent variables. The latent class (categorical) variable is used to model the unknown population heterogeneity and has the purpose of identifying clusters of individuals. The continuous latent variables (factors), on the other hand, account for the observed covariation among the test items within the classes and serve to capture the common content shared by the items. Because the common factor model can be obtained by setting the number of latent classes to 1, factor mixture models may be seen as a generalization of the common factor model. Alternatively, FMA may also be seen as a generalization of the classic latent class model, which can be derived by setting the factor variances to 0 in each class.

**FIGURE 1:**
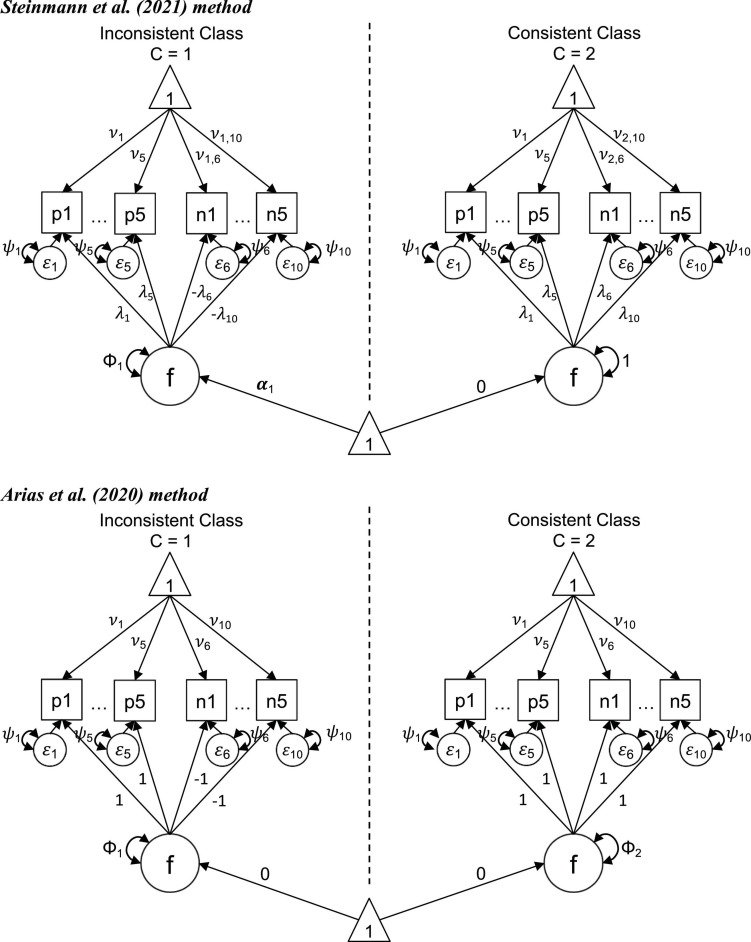
Constrained factor mixture analysis models for the Rosenberg Self-Esteem Scale. p1–p5, positively worded items; n1–n5, recoded negatively worded items; f, factor; ε, residual; ν, item intercept; λ, item factor loading; ψ, item uniqueness; Φ, factor variance; and α, factor mean.

A constrained FMA is an FMA that posits specific theoretical constrains on the parameter estimates. Constrained FMAs can be particularly useful to identify classes of consistent and inconsistent respondents to mixed-worded scales. Two recent constrained FMAs have been proposed to study population heterogeneity related to wording effects, Steinmann et al. (2021) and Arias et al. (2020). Both of these models specify a categorical latent variable with two latent classes (*k*), one for the inconsistent respondents (*k* = 1) and one for the consistent respondents (*k* = 2). We follow the graphical representation of the factor mixture model provided in Steinmann et al. (2021) and present in Figure 1 the constraints specified for each model, considering that the negative items (i.e., reversed items) have been recoded.

The constrained FMA proposed by Steinmann et al. (2021) includes the following constraints (Figure 1, top panel). The model posits that the individuals from the two classes respond similarly to the positively worded items. With *i* indicating the item number, the factor loadings (λ) and intercepts (ν) of the positively worded items (+) are set to be equal across classes ($\lambda_{1,i}^{+}=\lambda_{2,i}^{+}$ and $\nu_{1,i}^{+}=\nu_{2,i}^{+}$). In order to set the poles of the latent scales, the factor loadings of the positively worded items are constrained to be positive ($\lambda_{k,i}^{+}$>0). Regarding the *recoded* negatively worded items (–), their loadings are also constrained to be positive for the consistent class ($\lambda_{2,i}^{-}$>0), as the positive and recoded negative items of a scale are expected to correlate positively according to substantive theory. Because inconsistent individuals respond as if the positive and negative items had the same polarity, the loadings for the negative items of this class are set to be of equal magnitude but with opposite sign to the loadings for the consistent class ($\lambda_{1,i}^{-}=-\lambda_{2,i}^{-}$). Regarding the intercepts of the negatively worded items, they are estimated freely in both classes ($\nu_{1,i}^{-}\text{and}\nu_{2,i}^{-}$). The model posits additional constraints to ensure identification, including: the metric of the latent factor for the consistent class (which serves as the reference class) is standardized, with its mean (α_2_) set to zero and its variance (Φ_2_) set to 1. Conversely, the mean and variance of the latent factor for the inconsistent class are freely estimated (α_1_,Φ_1_). Finally, and in order to ensure empirical identification, the model posits that the variances of the residual terms (ε) are equal across classes (ψ_1,*i*_ = ψ_2,*i*_).

The constrained FMA model proposed by Arias et al. (2020) is more restricted than the one of Steinmann et al. (2021; i.e., estimates fewer parameters) and it is based on the RIIFA model (Figure 1, bottom panel). In a RIIFA model with *recoded* negative items the method factor is specified so that the unstandardized loadings of the positive items are set to 1 and the unstandardized loadings of the negative items are set to −1, to account for the wording effects. Thus, the Arias et al. (2020) model specifies that for the inconsistent class the loadings on the factor follow this pattern ($\lambda_{1,i}^{+}=1,\lambda_{1,i}^{-}=-1$). For the consistent class, on the other hand, the factor loadings of the positive items are set to be the same to those of the inconsistent class ($\lambda_{2,i}^{+}=\lambda_{1,i}^{+}=1$), while the loadings of the recoded negative are also set to 1, which reflects the consistency in the responses to both sets of items ($\lambda_{2,i}^{-}=-\lambda_{1,i}^{-}=1$). In this FMA model the variances of the factor for both classes is freely estimated (Φ_1_andΦ_2_). Similarly to the Steinmann et al. model, in the Arias et al. model the variances of the residual terms (ε) are equal across classes (ψ_1,*i*_ = ψ_2,*i*_). Conversely, whereas the Steinmann et al. (2021) model has different item intercepts across classes for the negatively worded items, in the Arias et al. model the item intercepts of both the positive and negative items are set to be equal across classes ($\nu_{1,i}^{+}=\nu_{2,i}^{+}and\nu_{1,i}^{-}=\nu_{2,i}^{-}$) to focus the differences between the classes solely on the direction of the item loadings. Also, the Arias et al. model posits that the factor means are set to zero for both classes (α_1_ = α_2_ = 0), indicating that the wording effects are not related to the trait levels on the substantive factor.

Arias et al. (2020) tested the screening capability of their constrained FMA on a subset of Emotional Stability, Extraversion, and Conscientiousness items from the Big Five personality markers (Goldberg, 1992). In their sample composed of adults between 18 and 75 years (*M* = 34.7, SD = 11.7) they found that the inconsistent class constituted between 4.4 to 10% of the sample. Also when these cases were removed from the sample the wording factors practically disappeared and the trait estimates became notably more accurate. On the other hand, Steinmann et al. (2021) tested their method in samples from young children and adolescents from three countries (Germany, Australia, and the United States) and four mixed-worded scales, including the RSES and math and reading self-concept scales. Their results indicated that the inconsistent class comprised between 7 and 20% of the samples. Additionally, they found that inconsistent respondents were poorer readers and had lower cognitive reasoning scores. Although Steinmann et al. (2021) suggested the possibility of running the factor analyses on the cleaned data (with the inconsistent respondents removed), they did not do so in their study.

### The Rosenberg Self-Esteem Scale

Self-esteem is an important concept in psychology and has been associated with several indicators of mental health. Having high self-esteem has been associated with multiple benefits such as: greater psychological well-being, as well as better social, work, and academic performance (Naranjo Pereira, 2007; Kuster et al., 2013; Nwankwo et al., 2015). Conversely, low self-esteem has been linked to the development of maladaptive behaviors, such as substance abuse, depression, anxiety, and poorer performance in educational and professional settings (Ortega and Del Rey, 2001; Góngora and Casullo, 2009; Orth et al., 2016; Mustafa et al., 2015; Ntemsia et al., 2017).

One of the most widely used measures for assessing self-esteem is the RSES (Rosenberg, 1965). Originally, Rosenberg (1965) considered self-esteem as a unitary construct that reflected individual differences in the assessment of self-esteem and self-respect. However, more than 50 years of research and hundreds of empirical studies have attempted to resolve the dispute over the dimensionality of the RSES (Gnambs et al., 2018). Although the scale was originally developed as a 10-item one-dimensional balanced scale with five positively worded and five negatively worded items, some of the early researchers found through exploratory factor analysis that the two groups of items formed two separate factors rather than a single theoretical factor of self-esteem (Carmines and Zeller, 1979). These two factors have been labeled as self-deprecation and self-confidence (Owens, 1993), self-deprecation and positive self-worth (Owens, 1994), and positive and negative self-evaluation, with global self-esteem as a second-order level factor (Roth et al., 2008).

While the majority of the RSES factor-analytic studies have rejected the unidimensionality of its item scores, a large body of research has converged on the interpretation of a substantive factor of self-esteem and one or two method factors related to wording effects (e.g., Tomás and Oliver, 1999; Corwyn, 2000; DiStefano and Motl, 2006, 2009; Gana et al., 2013; Supple et al., 2013; Wu et al., 2017). Additionally, many of these studies have concluded that the biased responses are contained primarily in the negatively worded items (Marsh, 1986; Tomás and Oliver, 1999; Corwyn, 2000; Quilty et al., 2006; Supple et al., 2013). Furthermore, numerous studies have tried to understand the nature of these wording effects, with diverse findings linking it to reading and reasoning ability, right amygdala volume, personality, anxiety, and ethnocultural differences (Marsh, 1986; Horan et al., 2003; DiStefano and Motl, 2006, 2009; Quilty et al., 2006; Marsh et al., 2010b; Supple et al., 2013; Tomás et al., 2013; Wang et al., 2015; Michaelides et al., 2016; Gnambs and Schroeders, 2020). Indeed, authors have argued that if these wording effects are not accounted for in the factor models they might confound and bias findings related to structural relationships, measurement invariance, latent mean comparisons, among others (Marsh et al., 2010b; Supple et al., 2013; Tomás et al., 2015). It is important to note, however, that recent research has questioned the interpretability of these wording method factor scores and their relationships with other variables (Nieto et al., 2021; Steinmann et al., 2021).

### The Present Study

The main objective of the current study was to examine the capability of two constrained FMA methods, Steinmann et al. (2021) and Arias et al. (2020), in screening the RSES scores for wording effects. A second objective was to use the screened data from the best performing FMA method to evaluate for the first time the psychometric properties of RSES scores for the Dominican Republic population. Because The RSES has been the most widely used scale to study wording effects for scales with mixed-worded items (Steinmann et al., 2021), we hypothesized that the data from the Dominican population would also be contaminated by this response bias. As the constrained FMA methods proposed by Steinmann et al. (2021) and Arias et al. (2020) are very recent, their performance had not been compared previously. Additionally, Steinmann et al. (2021) suggested but did not directly test the screening capability of their method. Further, it was important to test their performance on different sets of data, particularly those from a different culture (Steinmann et al., 2021).

To test the screening capability of the constrained FMA models we examined three criteria (Arias et al., 2020; Steinmann et al., 2021): (1) the unidimensionality of the screened data according to parallel analysis (Horn, 1965) and the scree test (Cattell, 1966), (2) the improvement in fit of the one-factor model for the screened data in comparison to the fit for the total sample, and (3) the comparison between the screened samples and the total sample in the fit and structure of alternative factor models (one-factor, two-factor CFA, and a CFA-RIIFA). Better screening performance was evidenced by the similarity in fit between the one-factor model and the multidimensional models, higher correlation between the positive and negative factors of the CFA, and lower method factor loadings in the CFA-RIIFA. After the optimal screening method was established, we used this screened sample to further validate the RSES scores for the Dominican population. For this, we performed analyses of measurement invariance, internal consistency reliability, and an evaluation of the relationships of self-esteem with psychological clinical diagnosis, sex, and age.

## Materials and Methods

### Participants

The sample for this study was composed of 632 participants from the Dominican Republic. Of these, 594 belonged to a community sample and were selected via a non-probabilistic snowball sampling strategy, and the remaining 38 were a convenience sample from a mental health center and had been diagnosed with a psychological clinical disorder. Of the sample, 41.9% (265) were men and 58.1% (367) were women. The mean age was 29.60 (SD = 10.28) years. In terms of the highest education level achieved, 1.9% (12) reported to have finished primary school, 22.9% (145) had finished high school, and 75.2% (475) had finished undergraduate university studies.

### Measures

#### Rosenberg’s Self-Esteem Scale

Self-esteem was measured using RSES (Rosenberg, 1965). This balanced scale measures a favorable or unfavorable attitude toward the self and it comprises 10 items, five positively worded and five negatively worded. The Spanish version of the RSES was used for this research (Echeburúa, 1995). The items were responded in paper and pencil form via a 4-point Likert scale from 1 (*strongly disagree*) to 4 (*strongly agree*). In a 53 nation study by Schmitt and Allik (2005) the scores of the RSES obtained a high mean alpha internal consistency reliability of 0.81.

### Procedure

In the first instance, a pilot study was conducted with a group of 30 people (58% women and 42% men), who were not included in the final sample. This sample was examined by means of an unstructured interview to determine whether there were any difficulties in the comprehension of the items that could affect the understanding of the questionnaire. No comprehension problems were observed and therefore no modifications were made to the items. Subsequently, data collection was started on the definitive sample. All participants were adequately informed of the research objectives, the anonymity of their responses, and their voluntary participation. Likewise, it was clarified that participation would not cause any harm and that they could leave the study whenever they wished. International ethical guidelines for studies with human beings were taken into account (American Psychological Association, 2017). After the participants gave their informed consent, the team of psychologists, members of the research group and trained for the application and correction of the RSES, administered the instrument in person. Subsequently, the data were tabulated and statistically processed.

### Statistical Analyses

#### Data Screening With Factor Mixture Analysis

The data was screened for wording effects using two constrained FMA procedures: Steinmann et al. (2021) and Arias et al. (2020). The M*plus* syntaxes for these analyses are included in Appendix Tables A1, A2. Due to the expected low prevalence of inconsistent response profiles (≈10%), and the initial sample size of 632, the total sample for the inconsistent latent class was expected to be small (<100 cases). Such sample sizes are low for a categorical treatment of the variables, which in the case of the FMAs would also require numerical integration. Because of this, and due to the fact that the objective of these analyses was solely to identify inconsistent respondents to the positively and negatively worded items, the FMAs were estimated treating the items as continuous and using a robust maximum likelihood estimator (MLR). The agreement between the classifications of two FMA methods was assessed with the kappa statistic, with values ≤ 0 indicating no agreement, 0.01–0.20 as none to slight, 0.21–0.40 as fair, 0.41– 0.60 as moderate, 0.61–0.80 as substantial, and 0.81–1.00 as almost perfect agreement (McHugh, 2012).

#### Dimensionality Assessment

The determination of the latent dimensionality underlying the RSES scores was assessed with parallel analysis (Horn, 1965), one of the most accurate methods available (Garrido et al., 2013, 2016; Lim and Jahng, 2019; Golino et al., 2020). According to the parallel analysis method, factors should be retained as long as their eigenvalues are greater than those from uncorrelated variables in the population. As recommended in the factor analytic literature, parallel analysis was interpreted in conjunction with Cattell’s scree test (Hayton et al., 2004). Parallel analysis was performed with the specifications recommended by Garrido et al. (2013) for ordinal variables: polychoric correlations, eigenvalues from the full correlation matrix, random permutations of the empirical data to generate the artificial datasets, and the mean criterion to aggregate the generated eigenvalues. A total of 1,000 artificial datasets were generated.

#### Factor Modeling Specifications

Confirmatory factor analysis (CFA) and structural equation models (SEM) were estimated using the weighted least squares with mean- and variance-adjusted standard errors estimator over polychoric correlations, a widely recommended estimator for categorical variables (Rhemtulla et al., 2012). The RIIFA model was employed to account for wording effects in the total sample (Maydeu-Olivares and Coffman, 2006). As the negative items had been recoded, their loading on the method factor in the CFA-RIIFA model were −1, while the loadings for the positive items were +1. The method factor was posited to be uncorrelated to the substantive factor in order to identify the model.

#### Fit Criteria

The fit of the non-mixture factor models was assessed with four complimentary indices: the comparative fit index (CFI), the Tucker-Lewis index (TLI), the root mean square error of approximation (RMSEA), and the standardized root mean square residual (SRMR). Values of CFI/TLI greater than or equal to 0.90 and 0.95 have been suggested are reflecting acceptable and excellent fits to the data, while values of RMSEA less than 0.08 and 0.05 as indicating reasonable and close fits to the data, respectively (Marsh et al., 2004; Marsh et al., 2010a). In the case of the SRMR index, values less than or equal to 0.05 would suggest good fit (Schermelleh-Engel et al., 2003), while values less than 0.08 can be considered as acceptable (Hu and Bentler, 1999; Schreiber et al., 2006). However, because the values of these fit indices are also affected by incidental parameters not related to the size of the misfit (Garrido et al., 2016; Beierl et al., 2018; Shi et al., 2018; Xia and Yang, 2018), they should not be considered golden rules, and must be interpreted with caution (Marsh et al., 2004; Greiff and Heene, 2017). Local fit was also examined with the modification indices and the standardized expected parameter change (SEPC) statistic (Saris et al., 2009; Whittaker, 2012). SEPCs above 0.20 in absolute have been suggested as salient (Whittaker, 2012).

#### Measurement Invariance

Analyses of factorial invariance were performed across sex and age according to four sequential levels of measurement invariance (Marsh et al., 2014): (a) configural invariance, (b) metric (weak) invariance, (c) scalar (strong) invariance, and (d) residual (strict) invariance. It was considered that the invariance level was supported if the fit for the more restricted model, when compared to the configural model, did not decrease by more than 0.01 in CFI or increase by more than 0.015 in RMSEA (Chen, 2007). The delta parameterization was used for the configural, metric, and scalar models, while the theta parameterization was used to test for residual invariance. Because the analyses for measurement invariance for categorical indicators require that items have observed responses to the same response options across groups, the first two categories for the positive items p1 and p2 were merged (see Table 1). Prior to the merging of these categories, the first response option for these two items was only selected for one of the groups being compared.

**TABLE 1:** Polychoric correlations between the RSES items.

*Sample*/Item*	p1	p2	p3	p4	p5	n1	n2	n3	n4	n5
**Total sample (*N* = 632)**										
p1. Person of worth	–									
p2. Have good qualities	0.67	–								
p3. Do things as well as others	0.44	0.70	–							
p4. Positive self-attitude	0.51	0.57	.052	–						
p5. Satisfied with self	0.36	0.46	0.42	0.70	–					
n1. Not much to be proud of	0.26	0.30	0.26	0.36	0.34	–				
n2. I’m a failure	0.43	0.49	0.41	0.46	0.41	0.62	–			
n3. I don’t respect myself	0.32	0.41	0.36	0.47	0.35	0.45	0.49	–		
n4. I feel useless at times	0.49	0.54	0.50	0.57	0.49	0.53	0.74	0.50	–	
n5. I am not a good person	0.41	0.41	0.22	0.43	0.32	0.41	0.48	0.47	0.55	–
**Screened sample with Steinmann et al. method (*N* = 588)**										
p1. Person of worth	–									
p2. Have good qualities	0.66	–								
p3. Do things as well as others	0.48	0.72	–							
p4. Positive self-attitude	0.53	0.59	0.53	–						
p5. Satisfied with self	0.38	0.51	0.44	0.71	–					
n1. Not much to be proud of	0.24	0.32	0.30	0.40	0.37	–				
n2. I’m a failure	0.51	0.60	0.51	0.60	0.48	0.57	–			
n3. I don’t respect myself	0.34	0.43	0.37	0.50	0.38	0.44	0.47	–		
n4. I feel useless at times	0.51	0.61	0.57	0.66	0.54	0.48	0.75	0.52	–	
n5. I am not a good person	0.41	0.45	0.29	0.50	0.36	0.38	0.49	0.48	0.51	–
**Screened sample with Steinmann et al. method (*N* = 582)**										
p1. Person of worth	–									
p2. Have good qualities	0.66	–								
p3. Do things as well as others	0.41	0.67	–							
p4. Positive self-attitude	0.54	0.57	0.52	–						
p5. Satisfied with self	0.38	0.47	0.42	0.72	–					
n1. Not much to be proud of	0.37	0.44	0.38	0.45	0.43	–				
n2. I’m a failure	0.55	0.65	0.57	0.60	0.53	0.59	–			
n3. I don’t respect myself	0.39	0.51	0.45	0.52	0.40	0.41	0.47	–		
n4. I feel useless at times	0.57	0.67	0.61	0.67	0.56	0.52	0.72	0.51	–	
n5. I am not a good person	0.50	0.53	0.33	0.51	0.38	0.36	0.43	0.46	0.51	–

p1–p5, positive items; n1–n5, negative items recoded. *p* < 0.001 for all polychoric correlations.

*Scale items have been paraphrased and shortened.

#### Reliability Analyses

The internal consistency reliability of the RSES scores was evaluated with Green and Yang’s (2009) categorical omega coefficient. Unlike the ordinal alpha and omega coefficients (Gadermann et al., 2012), categorical omega as the takes into account the ordinal nature of the data to estimate the reliability of the observed scores, rather than the reliability underlying hypothetical scores (Chalmers, 2018). Thus, it is recommended for Likert-type item scores (Yang and Green, 2015; Viladrich et al., 2017). In order to provide common reference points with the previous literature, Cronbach’s alpha, with the items treated as continuous, were also computed and reported. For all coefficients 95% confidence intervals were computed across 1,000 bootstrap samples using the bias-corrected and accelerated approach (Kelley and Pornprasertmanit, 2016).

#### Analysis Software

Data handling and classification agreement with the kappa statistic was performed using the IBM SPSS software version 25. Polychoric correlations, factor mixture models, CFA, and SEM models were estimated with the M*plus* program version 8.3. Dimensionality assessment with parallel analysis was conducted with the R function *fa.parallel* contained in the *psych* package (version 1.9.12.31; Revelle, 2020). Internal consistency reliability with the categorical omega and alpha coefficients was estimated with the *ci.reliability* function contained in the *MBESS* package (version 4.6.0; Kelley, 2019). Finally, Welch’s *t*-test and Cohen’s *d* measure of effect size (Cohen, 1992) were computed using the *jamovi* program version 1.6.23.0.

## Results

The results of this study were divided into two sections: (1) data screening with FMA, and (2) scale validation with the screened data. The first section included the estimates of the factor mixture models, as well as dimensionality assessments and factor model comparisons to evaluate the screening capability of the two FMA methods. The second section was based on the screened sample obtained from the best performing FMA, and its objective was to further validate the RSES scores for the Dominican population through tests of factorial invariance across sex and age, internal consistency estimates, and an evaluation of the relationships of self-esteem with psychological clinical diagnosis, sex, and age.

### Data Screening With Factor Mixture Analysis

#### Factor Mixture Analysis Estimates

The Steinmann et al. method replicated the best loglikelihood of −5,950.26 with 20,000 initial stage random starts and 5,000 final stage optimizations. A total of 44 cases (7.0%) were assigned to the first “inconsistent” latent class, while 588 cases (93.0%) were assigned to the second “consistent” latent class. These results indicate that only a small proportion of respondents responded considerably inconsistently to the positive and negative items. In the first class the standardized loadings (intercepts) for the five positive items were 0.38 (3.78), 0.51 (3.70), 0.47 (3.54), 0.62 (3.47), and 0.47 (3.31), while the loadings for the recoded negative items were −0.33 (2.04), −0.55 (1.59), −0.38 (2.06), −0.60 (2.22), and −0.40 (2.60). For the second class, the standardized loadings (intercepts) for the five positive items were 0.50 (3.78), 0.65 (3.70), 0.60 (3.54), 0.75 (3.47), and 0.60 (3.31), while the loadings for the negative items were 0.45 (3.30), 0.68 (3.66), 0.51 (2.95), 0.73 (3.49), and 0.52 (3.35). Regarding the classification quality, the entropy index for the solution was 0.97.

Regarding the Arias et al. method, with 20,000 initial stage random starts and 5,000 final stage optimizations, the best loglikelihood of −6,141.31 for the two-class factor mixture model was consistently replicated. A total of 50 cases (7.9%) were assigned to the first “inconsistent” latent class, while 582 cases (92.1%) were assigned to the second “consistent” latent class. Again, only a small proportion of respondents responded considerably inconsistently to the positive and negative items. In the first class the standardized loadings (intercepts) for the five regular items were 0.65 (3.74), 0.70 (3.66), 0.58 (3.49), 0.56 (3.42), and 0.47 (3.26), while the loadings for the recoded negative items were −0.40 (3.21), −0.51 (3.52), −0.36 (2.89), −0.50 (3.40), and −0.42 (3.29). For the second class, the standardized loadings (intercepts) for the five regular items were 0.74 (3.74), 0.78 (3.66), 0.67 (3.49), 0.65 (3.42), and 0.56 (3.26), while the loadings for the negative items were 0.49 (3.21), 0.60 (3.52), 0.45 (2.89), 0.59 (3.40), and 0.50 (3.29). In terms of the classification quality, the entropy index for the solution was 0.57.

We also computed the agreement between the classifications of the two FMA methods. According to the kappa coefficient of 0.33 (*p* < 0.001), there was only a fair level of agreement between the classifications of the two FMA methods.

#### Dimensionality Assessment With Parallel Analysis

The sample matrices of polychoric correlations for the total and screened samples are shown in Table 1. For the total sample the average correlations were 0.54 between the positive items, 0.53 between the recoded negative items, and 0.40 between the positive-negative item pairs. For the screened sample with the Steinmann et al. method the average correlations were 0.55 between the positive items, 0.51 between the negative items, and 0.45 between the positive-negative item pairs. Regarding the screened sample with the Arias et al. method, the average correlations were 0.54 between the positive items, 0.50 between the negative items, and 0.50 between the positive-negative item pairs. As can be seen, the correlations between the positive-negative item pairs for the total sample were notably lower than those between the positive items or between the negative items. These average correlations for the positive-negative item pairs increased by 0.05 for the Steinmann et al. screened sample, and by 0.10 for the Arias et al. screened sample, where they were closest to the average correlations for the positive and negative item groups.

**FIGURE 2:**
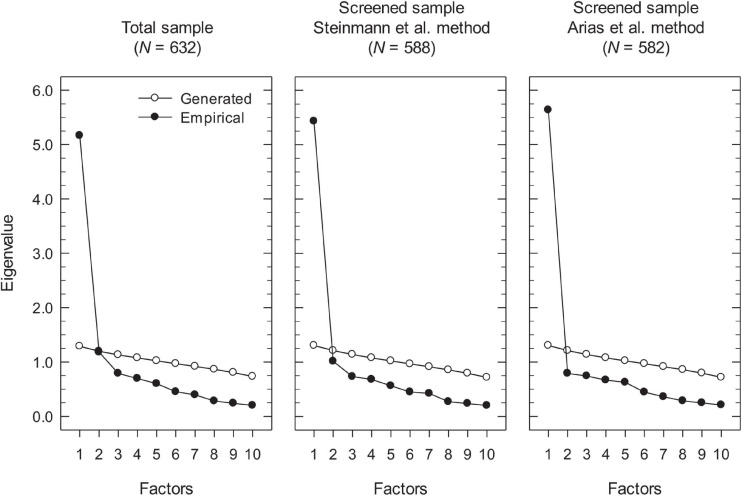
Dimensionality assessment of the RSES scores with parallel analysis.

The results obtained with the parallel analysis method for the total and screened samples are shown in Figure 2. Regarding the results for the total sample, the first factor had a large eigenvalue of 5.17 that was much higher than its random counterpart (1.29), while the second factor had an eigenvalue of 1.18 that was slightly lower than the eigenvalue of 1.20 for its random counterpart. Although according to these results parallel analysis suggested that only one factor be retained, a look at the eigenvalue plots in Figure 2 reveals that for the total sample there is a clear break starting with the third eigenvalue, a strong indication that the underlying structure is bidimensional rather than unidimensional. In the case of the of the Steinmann et al. screened data, the first eigenvalue was higher (5.43) and the second eigenvalue lower (1.01) than for the total sample, suggesting a more unidimensional structure. According to parallel analysis, the second factor should not be retained as its eigenvalue was sensibly lower than its random counterpart (1.21). However, the eigenvalue plot in Figure 2 still shows a break (albeit less sharp than for the total sample) starting at the third eigenvalue, suggesting that there was some systematic variance in the data beyond that of the first factor. In contrast, the results for the screened data with the Arias et al. method revealed a clear unidimensional structure, with a stronger first factor (eigenvalue of 5.64), and a noticeably weaker second factor with an eigenvalue of 0.79 that was much lower than the eigenvalue of 1.21 of its random counterpart. Additionally, the eigenvalue plot showed a very strong and clear break starting at the second eigenvalue, which supports the essential unidimensionality of the data.

#### Model Fit and Factorial Structures for the RSES Models

**FIGURE 3:**
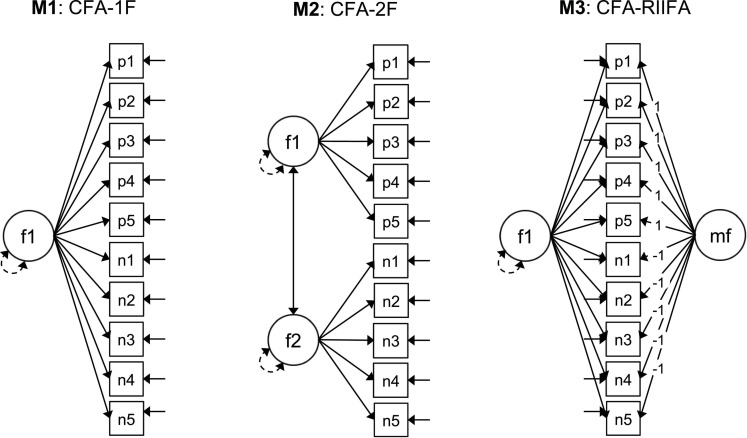
Factorial models examined for the RSES scores. CFA, confirmatory factor analysis; #F, number of substantive factors; RIIFA, random intercept item factor analysis; f1, f2, substantive factors; mf, method factor; p1–p5, positive items; n1–n5, negative items recoded. Squares represent observed variables. Circles represent latent variables. Unidirectional arrows linking circles and rectangles represent factor loadings. Bidirectional arrows linking the circles represent the factor covariances and correlations. Bidirectional arrows connecting a single circle represent the factor variances. Full unidirectional arrows linked to the squares represent item uniquenesses.

In order to further evaluate the screening capability for wording effects of the two FMA methods we compared the model fit and factorial structures for three RSES models (Figure 3): a one-factor model (CFA-1F), and two-factor confirmatory model where the positive and negative items were posited to load on separate correlated factors (CFA-2F), and a RIIFA model that posited a method factor for the wording effects (CFA-RIIFA). The criteria for screening quality were the improvements in fit for the CFA-1F model in the screened samples in comparison to the fit for the total sample, as well as the achieved similarity in fit between the CFA-1F model and the CFA-2F/CFA-RIIFA models. Additionally, we also estimated these factor models with the error correlation between items p4 “*I take a positive attitude toward myself*” and p5 “*On the whole, I am satisfied with myself*” freed (θ). According to the local fit statistics, this error correlation produced the highest modification index for all the models in the total sample and the sample screened with the Arias et al. method, and the first or second highest for the screened sample with the Steinmann et al. method. For example, in the case of the Arias et al. screened sample, the modification indices for the error correlation between the items p4 and p5 ranged between 70.78 and 84.51, while the second highest modification indices ranged between 18.09 and 24.36, making the former more than three times higher than the latter. Regarding the SEPC values for this error correlation, they ranged between 0.48 and 0.67 across models and samples, indicating a high level of local misfit for all cases.

The fit of the RSES factor models are presented in Table 2 and the factor solutions in Table 3. As can be seen in Table 2, the fit improved notably when adding the error correlation between items p4 and p5 for all models and samples. Additionally, Table 3 shows that the standardized estimates for this error correlation were considerably large, ranging between 0.38 and 0.51. Therefore, for simplicity, the commentary of the results will focus on the models that included this error correlation. As expected, the fit of the CFA-1F-θ for the total sample was not satisfactory (e.g., RMSEA = 0.128), with the CFA-2F-θ (e.g., RMSEA = 0.073), and CFA-RIIFA-θ models (e.g., RMSEA = 0.077) providing substantially better levels of it. Additionally, the factor solutions (Table 3) reveal that the correlation between the positive and negative factors in the CFA-2F-θ model was 0.77, which implies a shared variance of only 59.3%, suggesting a clear bidimensionality. In the case of the CFA-RIIFA-θ model, the loadings on the method factor were 0.25, indicating considerable wording effects.

**TABLE 2:** Fit statistics for the RSES factorial models.

S*ample*/Model	χ^2^	*df*	CFI	TLI	SRMR	RMSEA (90% C.I.)
**Total sample (*N* = 632)**						
CFA-1F	398.496*	35	0.904	0.877	0.067	0.128 (0.117, 0.140)
CFA-1F-θ	283.330*	34	0.934	0.913	0.061	0.108 (0.096, 0.119)
CFA-2F	191.319*	34	0.959	0.945	0.046	0.086 (0.074, 0.098)
CFA-2F-θ	144.485*	33	0.971	0.960	0.040	0.073 (0.061, 0.086)
CFA-RIIFA	199.980*	34	0.956	0.942	0.045	0.088 (0.076, 0.100)
CFA-RIIFA-θ	155.196*	33	0.968	0.956	0.040	0.077 (0.065, 0.089)
**Screened sample with the Steinmann et al. method (*N* = 588)**						
CFA-1F	253.613*	35	0.942	0.926	0.052	0.103 (0.091, 0.115)
CFA-1F-θ	178.568*	34	0.962	0.950	0.047	0.085 (0.073, 0.098)
CFA-2F	162.427*	34	0.966	0.955	0.042	0.080 (0.068, 0.093)
CFA-2F-θ	126.305*	33	0.975	0.966	0.038	0.069 (0.057, 0.082)
CFA-RIIFA	158.358*	34	0.967	0.957	0.041	0.079 (0.067, 0.091)
CFA-RIIFA-θ	124.260*	33	0.976	0.967	0.037	0.069 (0.056, 0.082)
**Screened sample with the Arias et al. method (*N* = 582)**						
CFA-1F	181.566*	35	0.964	0.953	0.042	0.085 (0.073, 0.097)
CFA-1F-θ	99.373*	34	0.984	0.979	0.033	0.057 (0.045, 0.071)
CFA-2F	165.677*	34	0.967	0.957	0.042	0.082 (0.069, 0.094)
CFA-2F-θ	97.527*	33	0.984	0.978	0.033	0.058 (0.045, 0.071)
CFA-RIIFA	166.569*	34	0.967	0.957	0.042	0.082 (0.070, 0.094)
CFA-RIIFA-θ	97.949*	33	0.984	0.978	0.033	0.058 (0.045, 0.072)

χ^2^, chi-square; *df*, degrees of freedom; CFI, comparative fit index; TLI, Tucker-Lewis index; SRMR, standardized root mean square residual; RMSEA, root mean square error of approximation; C.I., confidence interval; CFA, confirmatory factor analysis; #F, factors; RIIFA, random intercept item factor analysis; and θ, error correlation between items p4 and p5.*p < 0.001.

**TABLE 3:** Factorial loadings, error correlations, and factor correlations for the RSES models.

*Sample*	CFA-1F		CFA-2F			CFA-RIIFA		
Item*/Error correlation/Factor	F1	*h* ^2^	F1	F2	*h* ^2^	F1	MF	*h* ^2^
**Total sample (*N* = 632)**								
p1. Person of worth	**0.66**	0.43	**0.70**	*0.00*	0.49	**0.66**	0.25	0.49
p2. Have good qualities	**0.79**	0.62	**0.84**	*0.00*	0.71	**0.78**	0.25	0.67
p3. Do things as well as others	**0.66**	0.43	**0.71**	*0.00*	0.50	**0.66**	0.25	0.50
p4. Positive self-attitude	**0.69**	0.48	**0.77**	*0.00*	0.60	**0.73**	0.25	0.60
p5. Satisfied with self	**0.56**	0.32	**0.63**	*0.00*	0.39	**0.60**	0.25	0.43
n1. Not much to be proud of	**0.62**	0.38	*0.00*	**0.65**	0.42	**0.60**	−0.25	0.42
n2. I’m a failure	**0.80**	0.64	*0.00*	**0.83**	0.68	**0.78**	−0.25	0.67
n3. I don’t respect myself	**0.63**	0.39	*0.00*	**0.65**	0.43	**0.62**	−0.25	0.45
n4. I feel useless at times	**0.84**	0.71	*0.00*	**0.88**	0.78	**0.83**	−0.25	0.76
n5. I am not a good person	**0.61**	0.37	*0.00*	**0.64**	0.40	**0.60**	−0.25	0.42
θ	0.51		0.43			0.40		
F1	–		–			–		
F2/MF			0.77	–		*0.00*	–	
**Screened sample with the Steinmann et al. method (*N* = 588)**								
p1. Person of worth	**0.67**	0.44	**0.69**	*0.00*	0.47	**0.66**	0.20	0.48
p2. Have good qualities	**0.81**	0.66	**0.84**	*0.00*	0.71	**0.81**	0.20	0.69
p3. Do things as well as others	**0.70**	0.48	**0.72**	*0.00*	0.52	**0.69**	0.20	0.52
p4. Positive self-attitude	**0.76**	0.58	**0.81**	*0.00*	0.65	**0.79**	0.20	0.66
p5. Satisfied with self	**0.62**	0.38	**0.65**	*0.00*	0.42	**0.64**	0.20	0.44
n1. Not much to be proud of	**0.57**	0.32	*0.00*	**0.59**	0.34	**0.56**	−0.20	0.35
n2. I’m a failure	**0.82**	0.67	*0.00*	**0.84**	0.71	**0.81**	−0.20	0.70
n3. I don’t respect myself	**0.62**	0.39	*0.00*	**0.64**	0.41	**0.62**	−0.20	0.43
n4. I feel useless at times	**0.85**	0.72	*0.00*	**0.88**	0.77	**0.85**	−0.20	0.75
n5. I am not a good person	**0.61**	0.37	*0.00*	**0.63**	0.40	**0.61**	−0.20	0.41
θ	0.46		0.40			0.38		
F1	–		–			–		
F2/MF			0.87	–		*0.00*	–	
**Screened sample with the Arias et al. method (*N* = 582)**								
p1. Person of worth	**0.69**	0.48	**0.70**	*0.00*	0.49	**0.69**	0.07	0.49
p2. Have good qualities	**0.82**	0.68	**0.83**	*0.00*	0.68	**0.82**	0.07	0.68
p3. Do things as well as others	**0.70**	0.49	**0.70**	*0.00*	0.49	**0.70**	0.07	0.49
p4. Positive self-attitude	**0.76**	0.58	**0.77**	*0.00*	0.59	**0.76**	0.07	0.59
p5. Satisfied with self	**0.63**	0.40	**0.63**	*0.00*	0.40	**0.63**	0.07	0.40
n1. Not much to be proud of	**0.62**	0.39	*0.00*	**0.62**	0.39	**0.62**	−0.07	0.39
n2. I’m a failure	**0.82**	0.67	*0.00*	**0.82**	0.67	**0.82**	−0.07	0.67
n3. I don’t respect myself	**0.63**	0.40	*0.00*	**0.64**	0.41	**0.63**	−0.07	0.41
n4. I feel useless at times	**0.86**	0.74	*0.00*	**0.86**	0.74	**0.86**	−0.07	0.74
n5. I am not a good person	**0.62**	0.38	*0.00*	**0.62**	0.38	**0.62**	−0.07	0.38
θ	0.47		0.47			0.47		
F1	–		–			–		
F2/MF			0.98	–		*0.00*	–	

p1-p5, positive items; n1-n5, negative items recoded; CFA, confirmatory factor analysis; RIIFA, random intercept item factor analysis; #F, number of factors; F#, factor number; MF, method factor; *h*^2^, communality; and θ, error correlation for p4 and p5. Factor loadings ≥ 0.30 in absolute value appear bolded. All parameters are significant (*p* < 0.05). Parameters fixed to zero appear in italics.

*Scale items have been paraphrased and shortened.

Regarding the screened sample with the Steinmann et al. method, Table 2 shows that the fit of the CFA-1F-θ model was better (e.g., RMSEA = 0.085) than for the total sample, and closer to the fits of the CFA-2F-θ and CFA-RIIFA-θ models (e.g., RMSEA = 0.069). In addition, the factor correlation for the CFA-2F-θ model was 0.87, indicating a higher shared variance of 75.7%, while the loadings on the method factor were 0.20, lower than those for the total sample (Table 3). In all, these results indicate a reduction in the amount of wording effects in the data. At the same time, they show that these screened data still contained non-negligible systematic variance due to wording effects, a result that was in line with those of the dimensionality assessments. In terms of the screened sample with the Arias et al. method, the fit of the three models was approximately equal (e.g., 0.057 ≤ RMSEA ≤ 0.058), and better than the fit of all the models for the total sample or the Steinmann et al. screened sample, particularly for the CFA-1F-θ models (Table 2). Additionally, the correlation between the factors for the CFA-2F-θ model was almost perfect at 0.98 (96.0% shared variance), and the loadings on the method factor could be considered negligible at 0.07. These results indicate that the Arias et al. method was able to screen out almost all the wording effects variance in the data, making it the most effective FMA method. Furthermore, for this screened sample the underlying structure could be considered essentially unidimensional given the good fit of the CFA-1F-θ model (CFI = 0.984, TLI = 0.979, SRMR = 0.033, and RMSEA = 0.057 [90% C.I. = 0.045, 0.071]) and the results of the previous dimensionality assessments. Thus, the rest of the analyses were conducted on this sample.

#### Latent Class Comparisons on the RSES Scores and Sociodemographic Characteristics

We performed additional inferential analyses in order to better understand the differences between the responses and participants corresponding to the consistent and inconsistent latent classes obtained with the Arias et al. method. First, we computed mean scores for the positive and the recoded negative items separately and compared them across the groups corresponding to the two classes. In terms of the observed scores on the *positive* items, Welch’s *t*-test indicated that the inconsistent class (*M* = 3.56, SD = 0.424) and the consistent class (*M* = 3.55, SD = 0.448) had equal mean scores (*t*[58.8] = 0.129, *p* = 0.898, and *d* = 0.019). However, in the case of the *negative* item scores, the consistent class (*M* = 3.30, SD = 0.603) obtained significantly higher means than the inconsistent class (*M* = 2.96, SD = 0.897, *t*[52.9] = 2.667, *p* = 0.010, and *d* = 0.451). These results would appear to suggest that the response biases mainly affected the negative items. Second, we examined whether class membership was related to the *sex* and *age* of the participants. In terms of sex, Fisher’s exact test of independence indicated that sex was unrelated to latent class membership (*p* = 0.882). In contrast, the results of Welch’s *t*-test indicated that the participants in the inconsistent class (*M* = 33.14, SD = 11.496) were older than the participants in the consistent class (*M* = 29.30, SD = 10.121, *t*[55.7] = 2.290, *p* = 0.026, and *d* = 0.355).

### Scale Validation With the Screened Data

#### Measurement Invariance Across Sex and Age

The results for the factorial measurement invariance tests across sex (women, men) and age (18–29, 30–63) for the Arias et al. screened sample are presented next in Table 4. In each case, measurement invariance was evaluated for the CFA-1F-θ model at the configural, metric, scalar, and residual level. Regarding measurement invariance across sex, the results indicated that the configural model fit the data adequately (CFI = 0.979, TLI = 0.972, and RMSEA = 0.069). Moreover, none of the more stringent levels of measurement invariance produced sufficiently large deterioration of fit (i.e., ΔCFI ≤ 0.010, RMSEA ≥ 0.015) to reject the measurement invariance between men and women. Conversely, in some cases the fit of the more stringent invariance model was actually better than that of the configural model (e.g., RMSEA = 0.063 for the residual model, while RMSEA = 0.069 for the configural model). Therefore, measurement invariance across sex was supported at all levels. In terms of invariance across age, the results showed that the configural model also fit the data adequately (CFI = 0.983, TLI = 0.978, and RMSEA = 0.061). Similarly to sex, all the measurement invariance levels were supported for age, as the fit of the more stringent models were approximately equal or better than that of the configural invariance model.

**TABLE 4:** Factorial invariance of the RSES for the screened sample with the Arias et al. method (*N* = 582).

*Variable*	Overall model fit					Change in model fit				
Invariance Model	χ^2^	*df*	CFI	TLI	RMSEA	Δχ^2^	Δ*df*	ΔCFI	ΔTLI	ΔRMSEA
**Sex**										
Women (*N* = 337)	100.7*	34	0.976	0.968	0.076					
Men (*N* = 245)	62.1*	34	0.982	0.976	0.058					
M1. Configural (none)	161.0*	68	0.979	0.972	0.069					
M2. Metric (FL)	173.6*	77	0.978	0.974	0.066	21.7*	9	−0.001	0.002	−0.003
M3. Scalar (FL,Th)	182.9*	94	0.980	0.981	0.057	41.2*	26	0.001	0.009	−0.012
M4. Residual (FL,Th,Uniq)	225.1*	104	0.973	0.976	0.063	83.6*	36	−0.006	0.004	−0.006
**Age**										
18–29 (*N* = 366)	82.9*	34	0.979	0.972	0.063					
30–63 (*N* = 216)	62.4*	34	0.987	0.983	0.062					
M1. Configural (none)	142.7*	68	0.983	0.978	0.061					
M2. Metric (FL)	148.7*	77	0.984	0.981	0.057	18.6*	9	0.001	0.003	−0.004
M3. Scalar (FL,Th)	167.5*	94	0.984	0.984	0.052	42.7*	26	0.001	0.006	−0.009
M4. Residual (FL,Th,Uniq)	189.5*	104	0.981	0.984	0.053	67.5*	36	−0.002	0.006	−0.008

χ^2^, chi-square; *df*, degrees of freedom; CFI, comparative fit index; TLI, Tucker-Lewis index; RMSEA, root mean square error of approximation; M, measurement invariance model; FL, factor loadings; Th, thresholds; and Uniq, item uniquenesses; The parameters constrained to be equal across groups are shown in the parentheses next to the invariance models. The chi-square difference tests between nested models was conducted using M*plus’* DIFFTEST option. The metric, scalar, and residual invariance models were compared against the configural model. The invariance models were estimated using the Delta parameterization, except for the residual models where the Theta parameterization was required.

*p < 0.05.

#### Internal Consistency Reliability

According to the Green and Yang’s categorical omega coefficient, the scores of the RSES had a very high internal consistency reliability of 0.89 (95% C.I. = 0.87, 0.91). In order to provide a comparison point with previous studies we also computed the suboptimal alpha coefficient, which produced a reliability estimate of 0.85 (95% C.I. = 0.83, 0.88). In terms of the item-rest correlations, all items produced high values. The item-rest correlations for the positive items were 0.49 (p1), 0.62 (p2), 0.54 (p3), 0.68 (p4), and 0.56 (p5). In the case of the recoded negative items, the item-rest correlations were 0.49 (n1), 0.64 (n2), 0.51 (n3), 0.71 (n4), and 0.49 (n5).

#### Relationships of Self-Esteem With Psychological Clinical Diagnosis, Sex, and Age

**FIGURE 4:**
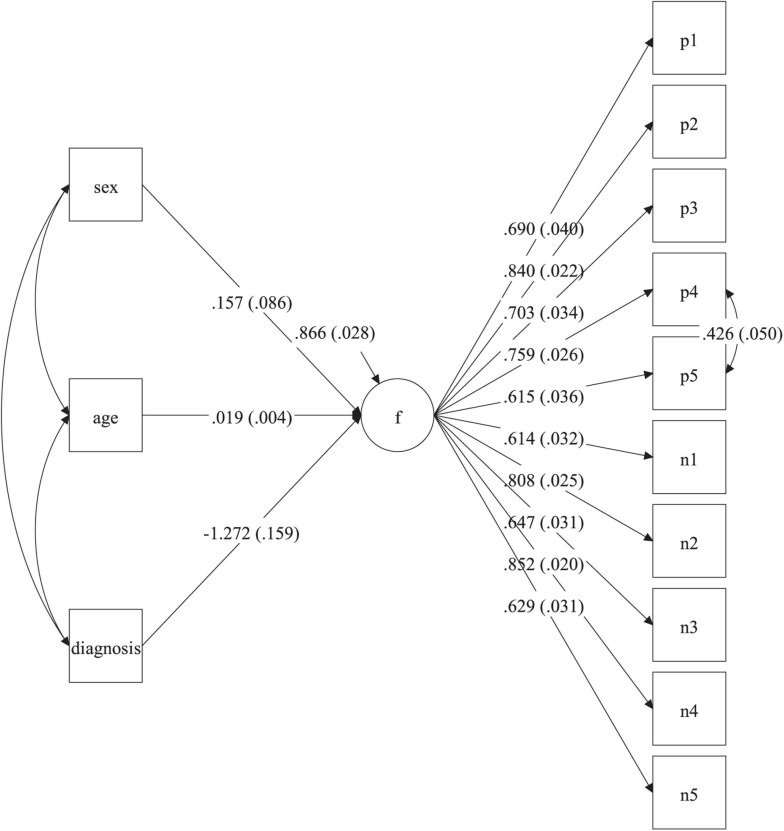
SEM MIMIC model for the prediction of self-esteem. f, self-esteem factor; p1–p5, positive items; n1–n5, recoded negative items; sex (0 = men, 1 = women); diagnosis, psychological diagnosis (0 = no, 1 = yes). Values in parenthesis indicate standard errors. Estimates correspond to the solution with the latent factor (f) standardized. *p* < 0.001 for all factor loadings and the age and diagnosis regression weights. *p* = 0.069 for the sex regression weight. *p* < 0.001 for the explained variance of the self-esteem factor.

The final analyses of the RSES scores consisted in the estimation of a SEM multiple indicator multiple cause (MIMIC) model to determine if psychological clinical diagnosis, sex, and age were related to self-esteem, while statistically controlling for the rest of the predictors (Figure 4). Regarding the fit of the SEM MIMIC model, it was good: χ^2^_(61)_ = 182.50 (*p* < 0.001), CFI = 0.967, TLI = 0.960, and RMSEA = 0.059 (90% C.I. = 0.049, 0.068). According to the estimated solution with the dependent latent variable (self-esteem factor) standardized (Figure 4), psychological clinical diagnosis had a significant effect of −1.27 (*p* < 0.001). This result implies that those with psychological clinical diagnosis had latent self-esteem scores that were 1.27 standard deviations lower than those who did not have a psychological clinical diagnosis. The variable age also had a significant effect of 0.02 (*p* < 0.001). In this case, this implies that for every increase of 1 year in age, the self-esteem latent scores of the participants increased by 0.02 standard deviations. In contrast, sex did not have a significant effect on self-esteem (0.16, *p* = 0.069).

## Discussion

Although self-report is one of the most common methods to collect information in the behavioral and health sciences (Weijters et al., 2010; Demetriou et al., 2015; Fryer and Nakao, 2020), its validity is often threatened by response biases, particularly the inconsistency in the responses to positively and negatively worded items of the same dimension (Weijters et al., 2013; Plieninger, 2017; Baumgartner et al., 2018; Chyung et al., 2018; Vigil-Colet et al., 2020). Researchers have traditionally accounted for these wording effects by adding one or multiple method factors in a CFA framework (Tomás and Oliver, 1999; Gnambs et al., 2018). However, this approach has some important limitations such as wrongly assuming population homogeneity, not being able to recover the uncontaminated trait scores of the inconsistent participants, and producing method factors that have a nature and meaning that is difficult to interpret (Nieto et al., 2021; Steinmann et al., 2021). Alternatively, researchers have recently proposed constrained FMAs models that can handle the population heterogeneity typical of data with wording effects. By making it possible to screen out the inconsistent respondents, these constrained FMAs offer advantages over the “method factor approach” that can potentially move the field forward. However, these methods are very recent and as such had not been tested extensively or directly compared against each other. Therefore, the first objective of this study was to test and compare the screening capability of the constrained FMA methods proposed by Steinmann et al. (2021) and Arias et al. (2020) with RSES data from the Dominican Republic. A second objective was to examine for the first time the psychometric properties of RSES for the Dominican population, and in the process show how using constrained FMAs can aid in this validation process.

### Main Findings

Initial analyses of the factor structure of the RSES for the Dominican population indicated that the unidimensional model did not fit the data adequately, and that the incorporation of a method factor in the model showed that there was considerable wording effects variance in the data. These results are in line with the large body of research of the RSES (Tomás and Oliver, 1999; Corwyn, 2000; DiStefano and Motl, 2006, 2009; Gana et al., 2013; Supple et al., 2013; Wu et al., 2017), which is the most studied psychological scale for wording effects (Steinmann et al., 2021).

The constrained FMAs of Arias et al. (2020) and Steinmann et al. (2021) identified between 7 and 8% of the participants as responding inconsistently to the positively and negatively worded items of the RSES. This amount of inconsistent profiles is in line with the previous studies of these methods, which found between 7 and 20% for children and adolescent populations (Steinmann et al., 2021), and between 4 and 10% for adults (Arias et al., 2020). Comparisons between the observed scores for the consistent and inconsistent groups revealed that the inconsistent respondents had lower observed scores on the negative items, but no mean differences were detected in the responses to the positive items. These results suggest that the response bias might be contained primarily in the responses to the negative items, a finding that is in line with a large amount of RSES studies (Marsh, 1986; Tomás and Oliver, 1999; Corwyn, 2000; Quilty et al., 2006; Supple et al., 2013). Additionally, inconsistent respondents were found to be older than consistent respondents, while sex was found to be unrelated to class membership.

Regarding the screening capability for wording effects of the two constrained FMAs, the results indicated that removing the inconsistent respondents identified by both FMAs reduced the amount of wording effects in the database. However, whereas the Steinmann et al. method only cleaned the data partially, the Arias et al. (2020) method was able to remove the great majority of the wording effects variance. This finding was based on comparisons of the total and screened samples on estimates of latent dimensionality, and three factorial models: one-factor CFA, two-factor CFA, and a CFA-RIIFA model. The performance of the Arias et al. (2020) method is in line with the results from their study, which found the method to perform very well in removing wording effects variance with data from Big Five personality scales. In contrast, Steinmann et al. (2021) suggested the possibility of using their method to screen out inconsistent responses, but did not directly test its performance for this purpose.

Another objective of this study was to assess for the first time the psychometric properties of the RSES for the Dominican population. Based on the screened sample with the Arias et al. (2020) method we found evidence of essential unidimensionality for the RSES scores (Slocum-Gori et al., 2009; Slocum-Gori and Zumbo, 2011), with generally high factor loadings across the ten items (mean loading of 0.71), and very high internal consistency reliability (0.89). Additionally, the RSES was able to distinguish between individuals with and without clinical psychological diagnosis (the latter having substantially lower self-esteem latent scores). Also, sex was found to be unrelated to the RSES latent scores, while the RSES self-esteem latent scores were positively related with age, a result that is in line with previous RSES studies (Marsh et al., 2010b). In all, the findings from this study indicate that the RSES scores are valid and reliable for the Dominican population.

### Limitations and Future Directions

There are some limitations in this study that should be noted. One limitation was treating the RSES scores as continuous for the estimation of the FMA models. Because the inconsistent class is typically a small proportion of the sample, with our current sample size it wasn’t optimal to use categorical variable estimation. Thus, for the FMAs we treated the variables as continuous, in line with Arias et al. (2020) and Steinmann et al. (2021). As the main objective of the FMAs for this study was to identify consistent and inconsistent respondents, treating the variables as continuous might not have impacted their performance in a meaningful way. Nevertheless, future studies are needed to assess which treatment of the variables would be optimal for wording effects screening purposes. Another limitation is that we tested the performance of the constrained FMA methods with one scale, the RSES. Although the RSES has been the most studied scale for wording effects (Steinmann et al., 2021), we recommend that these constrained FMA methods continue to be tested with other mixed-worded scales and with samples from different cultures in the future, to add to the body of evidence established in Arias et al. (2020); Steinmann et al. (2021), and the present study. Finally, we compared the classes of consistent and inconsistent respondents in terms of their sex and age. We recommend that other relevant variables, such as cognitive ability and personality, are included in future studies to compare these groups and potentially enrich our understanding of the nature of wording effects.

### Practical Implications

Incorporating response quality screening techniques is important to ensure that the results from the self-report are valid and interpretable. Thus, we recommend that researchers incorporate constrained FMAs into their toolbox and consider using them to screen out individuals who respond inconsistently to mixed-worded scales. Recent research has shown that while adding method factors can account for wording effects variance in the data, it cannot recover unbiased person scores of preserve structural relationships (Nieto et al., 2021). Therefore, cleaning the data by removing these invalid profiles might be a worthwhile alternative. Our findings indicate that the method proposed by Arias et al. (2020) is excellent for this task, and it is our first recommendation. Nevertheless, more studies are needed to determine its efficacy and that of the method proposed by Steinmann et al. (2021). Additionally, researchers can follow the framework and sequence of analyses presented in study to evaluate the screening capability for wording effects of the constrained FMAs.

The question arises as to what to do with the responses of individuals that are flagged as inconsistent by the constrained FMAs. If the researcher is interested in obtaining inferences about the population, we suggest that these response vectors of very poor quality be simply removed from the databases and the analyses of interest be conducted on the cleaned data. Additionally, researchers can study the characteristics of the individuals that are flagged as inconsistent to further the understanding of wording effects. Until now, a large body of research has examined the correlates of wording method factors for this objective, but this approach is questionable because the information contained in these method factors is inherently ambiguous due to its numerous underlying causes and can be very difficult to interpret (Nieto et al., 2021; Steinmann et al., 2021). Comparing the characteristics of consistent and inconsistent individuals, on the other hand, is straightforward and might advance our knowledge. On the other hand, if practitioners are interested in using a mixed-worded scales to make decisions about individuals, then we suggest that scores from individuals flagged as inconsistent not to be interpreted. The practitioner can ask the individual to respond to scale a second time, ask them to play close attention to the items, and see if they can produce an interpretable response profile. If that is not possible or the individual does not produce a different result, we recommend that the practitioner use another means of evaluation.

## Data Availability Statement

The raw data supporting the conclusions of this article will be made available by the authors, without undue reservation.

## Author Contributions

All authors listed have made a substantial, direct and intellectual contribution to the work, and approved it for publication.

## Conflict of Interest

The authors declare that the research was conducted in the absence of any commercial or financial relationships that could be construed as a potential conflict of interest.

## Publisher’s Note

All claims expressed in this article are solely those of the authors and do not necessarily represent those of their affiliated organizations, or those of the publisher, the editors and the reviewers. Any product that may be evaluated in this article, or claim that may be made by its manufacturer, is not guaranteed or endorsed by the publisher.

## Appendix A

### M*plus* Syntaxes of the Factor Mixture Analyses

**TABLE A1:** M*plus* Syntax for the Factor Mixture Analysis with the Steinmann et al. Method.

TITLE:	RSES factor mixture analysis with the Steinmann et al. method	
DATA:	File = RSES.dat;	
VARIABLE:	Names = sex age education diagnosis p1-p5 n1-n5;	
	Usevar = p1-p5 n1-n5;	!*p1-p5 = positive items, n1-n5 = negative items recoded*
	Classes = c(2);	!*Two latent classes are estimated*
ANALYSIS:	Type = mixture; Estimator = MLR; Starts = 20000 5000;	
	Process = 3 (starts);	!*Number of core processors to utilize*
MODEL:	%overall%	
	f by p1* p2-p5 (a1-a5)	!*Items load on a single factor and*
	n1-n5;	!*all the loadings are estimated*
	f@1;	!*Factor variance is fixed to 1 for identification*
	[f@0];	!*Factor mean is fixed to zero for identification*
	%c#1%	!*This is the INCONSISTENT class*
	f;	!*Factor variance is estimated for class1*
	[f];	!*Factor mean is estimated for class1*
	f by n1-n5 (b1-b5);	!*Negative items have class specific loadings*
	[n1-n5];	!*Negative items have class specific intercepts*
	%c#2%	!*This is the CONSISTENT class*
	f by n1-n5 (c1-c5);	!*Negative items have class specific loadings*
	[n1-n5];	!*Negative items have class specific intercepts*
MODEL CONSTRAINT:		
	DO (1, 5) a#>0;	!*Positive items are set to have positive loadings*
	DO (1, 5) b#<0;	!*Class1 negative items are set to have negative loadings*
	DO (1, 5) c#=-b#;	!*Class2 negative items are set to have loadings equal to* !*the negative items in class1, but with opposite sign*
OUTPUT:	standardized; tech7; tech12;	
SAVEDATA: File = FMA2CSteinmann.dat; Format = free; Save = cprobabilities;		

**TABLE A2:** M*plus* Syntax for the Factor Mixture Analysis with the Arias et al. Method.

TITLE:	RSES factor mixture analysis with the Arias et al. method	
DATA:	File = RSES.dat;	
VARIABLE:	Names = sex age education diagnosis p1-p5 n1-n5;	
	Usevar = p1-p5 n1-n5;	!*p1-p5 = positive items, n1-n5 = negative items recoded*
	Classes = c(2);	!*Two latent classes are estimated*
ANALYSIS:	Type mixture; Estimator = MLR; Starts = 2000 500;	
	Process = 3 (starts);	!*Number of core processors to utilize*
MODEL:	%overall%	
	f by p1-n5;	!*Items load on a single factor*
	[p1-n5](i1-i10);	!*Item intercepts are equal across classes*
	%c#1%	!*This is the INCONSISTENT class*
	f by p1-p5@1 n1-n5@-1;	!*Negative items have opposite sign (-1) loadings*
	f*;	!*Factor variance is estimated*
	[f@0];	!*Factor mean is fixed to zero for identification*
	%c#2%	!*This is the CONSISTENT class*
	f by p1-n5@1;	!*All items are have the same factor loading*
	f*;	!*Factor variance is estimated*
	[f@0];	!*Factor mean is fixed to be equal to class1*
OUTPUT:	standardized; tech7; tech12;	
SAVEDATA: File = FMA2CArias.dat; Format = free; Save = cprobabilities;		
